# Redo Coronary Artery Bypass Grafting in the era of Advanced PCI

**DOI:** 10.21470/1678-9741-2019-0206

**Published:** 2022

**Authors:** Ter-Er Kusu-Orkar, Kellan Masharani, Amer Harky, Andrew D Muir

**Affiliations:** 1 School of Medicine, University of Liverpool, Liverpool, United Kingdom.; 2 Department of Cardiothoracic Surgery, Liverpool Heart and Chest Hospital, Liverpool, United Kingdom.

**Keywords:** Percutaneous Coronary Intervention, Survival Rate, Coronary Artery Bypass, Coronary Artery Disease, Coronary Stenosis

## Abstract

**Objective:**

To review the evidence behind the role and relevance of redo coronary artery bypass grafting (CABG) in the current practice of percutaneous coronary intervention (PCI).

**Methods:**

A comprehensive electronic literature search was performed to identify articles that discuss the practice of PCI and redo CABG in patients that require coronary revascularization. All relevant studies are summarized in narrative manner to reflect current indications and preference.

**Results:**

The advancement in utilization of PCI has reduced the rate of redo CABG in patients with previous CABG that requires revascularization of an already treated coronary disease or a new onset of coronary artery stenosis. Redo CABG is associated with satisfactory perioperative outcomes but higher mortality at immediate postoperative period when compared to PCI.

**Conclusion:**

Redo CABG patients are less likely to develop comorbidities associated with revascularisation, but the operative mortality is higher and long-term survival rates are similar in comparison to PCI. There is a need for further research into the role of redo CABG in the current advanced practice of PCI.

**Table:** 

Abbreviations, acronyms & symbols				
ART	= Arterial revascularisation trial		LAD	= Left anterior descending artery
BIMA	= Bilateral internal mammary arteries		LDL	= Low-density lipoprotein
BMS	= Bare metal stent		LIMA	= Left internal mammary artery
CABG	= Coronary artery bypass grafting		LV	= Left ventricular
CTO	= Chronic total occlusions		OCT	= Optical coherance tomography
DAPT	= Dual antiplatelet therapy		PCI	= Percutaneous coronary intervention
DES	= Drug eluting stent		RA	= Radial artery
ECG	= Electrocardiography		RAPCO	= Radial Artery Patency and Clinical Outcomes
GEA	= Gastro-epiploic artery		RCT	= Randomised controlled trials
IMA	= Internal mammary artery		RIMA	= Right internal mammary artery
IVUS	= Intravascular ultrasound		SVG	= Saphenous vein graft

## INTRODUCTION

Redo coronary artery bypass grafting (CABG) had long been considered as the most effective method of revascularisation after a primary CABG^[^^1^^]^. This repeat operation is strongly indicated in patients with symptoms of cardiac ischaemia despite medical therapy and presence of a graftable coronary artery in a viable myocardial territory. The European Society of Cardiology and the European Association for Cardio-Thoracic Surgery guidelines for revascularisation outline strategies to treat graft failure following CABG^[^^2^^]^. Choice of percutaneous coronary intervention (PCI) or redo CABG is centred on the timing of graft failure and the extent of coronary disease in such patients. Nevertheless, redo CABG operations have seen a staggering decline over the last 20 years^[^^3^^]^. The Society of Thoracic Surgeons database illustrates a 4% decline in the number of CABGs performed relative to redo CABG; which now stands at 2%^[^^4^^,^^5^^]^. This trend is partially attributed to the increased use of arterial rather than venous conduits for primary CABG^[^^6^^]^. When comparing the left internal mammary artery (LIMA) to the previously favoured long saphenous vein graft (SVG), research continuous to demonstrate a significant advantage in the patency rates of LIMA at one, five, and 10 years post CABG^[^^7^^]^. In conjunction with a more aggressive use of antiplatelet medications, statins, and antihypertensive, patient survival after a primary CABG has increased; but this means that the need for revascularisation will inevitably increase as ageing population also increases. Notably, the mortality, morbidity, and risks of redo CABG such as operation failure, bleeding, and infection significantly increase with age^[^^8^^]^. One of the main operational risks involves the re-sternotomy itself, which is particularly difficult to navigate without complications. Skeletonised veins which adhere to the sternum and old SVGs can be easily damaged during sternotomy^[^^9^^]^. Furthermore, there is a higher risk of perioperative bleeding during redo CABG which should be taken into serious consideration when performing such procedure^[^^10^^]^. Alongside neurological complications, such as stroke, and an increased likelihood of failure to wean patients off cardiopulmonary bypass, redo CABG carries higher morbidity and mortality in elective, urgent, and emergency cases in comparison to a primary CABG^[^^11^^]^. Hence the involvement of a more minimally invasive procedure such as PCI has improved patient outcomes but precluded the use of redo CABG for many indications/patient circumstances^[^^2^^]^. This paper will review the use of redo CABG and explore its relevance in light of the increased use and beneficial outcomes of PCI.

## GRAFT PATENCY AND PREDISPOSITION TO GRAFT BLOCKS

**Fig. 1:**
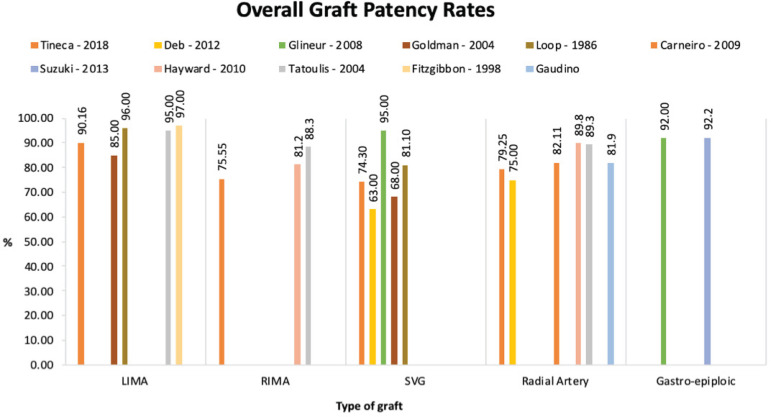
Patency rates of five different grafts types. LIMA=left internal mammary artery; RIMA=right internal mammary artery; SVG=saphenous vein graft

The decisive factor in determining the patency and predisposition to blockage of a CABG is the choice of conduit itself. Better understanding each graft and how best to utilise it is important for improved patient outcomes. Figure 1 is a summary of the key studies comparing graft outcomes and patency rates overtime^[^^7^^,^^12^^-^^20^^]^. LIMA is now the preferred conduit for a CABG. It demonstrates superior overall early, five- and 10-year patency rates in comparison to other conduit types. This is particularly true for anastomoses with the left anterior descending artery (LAD), to the extent that the overall patency in comparison to other grafts may be indifferent if not for this LAD-LIMA data^[^^7^^]^. Furthermore, LIMA has a relatively thin media that contains multiple elastic laminae and a lack of muscle incorporated in its structure. These features encourage its long-term patency as they reduce the tendency for the conduit to develop atherosclerosis or spasm^[^^21^^]^. This is in contrast to venous conduits, of which 75% are severely diseased or partially occluded at 10 years^[^^22^^]^. The right internal mammary artery (RIMA) also has superior patency rates to a SVG, and a large meta-analysis including 29,000 patients and 27 observational reports demonstrated that this vessel in combination with LIMA, a bilateral internal mammary arteries (BIMA) graft, significantly reduced long-term (nine years) patient mortality (hazard ratio, 0.78; confidence interval, 0.72-0.84; *P*<0.00001)^[^^7^^,^^23^^]^. These findings echo the trends in the arterial revascularisation trial (ART) at five and 10 years^[^^24^^,^^25^^]^. Nevertheless, ART did not find this trend significant, but it did find an increased risk of sternal complications and reconstruction associated with the use of BIMA grafts. Therefore, with some deviation to the recommendations given by the BIMA meta-analysis, more careful consideration should be taken when opting for this intervention.

The success of arterial grafts for revascularisation has inspired developments in the use of the radial artery (RA) and more novel conduits such as the Gastro-epiploic artery (GEA). RA has shown potential as a good second choice conduit to LIMA. A total of six randomised controlled trials (RCT) have compared RA grafts to SVGs and there has been some discordance in their findings^[^^12^^,^^26^^-^^30^^]^. The Radial Artery Patency and Clinical Outcomes (RAPCO) trial was the first single-centre trial comparing RA to RIMA and RA to SVG, although the differences weren’t significant, it was found that in both groups the RA patency at five years was lower (95% [RA] *vs*. 100% [RIMA], *P*=0.40 in group 1 and 87% [RA] *vs*. 94% [SVG], *P*= 0.50 in group 2)^[^^31^^]^. Following RCTs also failed to demonstrate any difference in these two conduits; nevertheless, the first multicentre RCT with a mean follow-up of 7.7 years demonstrated a significantly higher occlusion rate in SVG than in RA grafts (8.9% *vs*. 18.6%, *P*=0.002)^[^^30^^]^. These findings were also supported by a recent meta-analysis that demonstrated that a SVG was three times more likely to cause late functional graft occlusion when compared to the RA and four times more likely than a RIMA conduit^[^^32^^]^. Despite this, only one RCT carried out comparing RA and RIMA found no difference in patency rate and event-free survival rate at a 10-year follow-up^[^^17^^]^. The use of RA remains somewhat controversial; however, the current literature suggests it is a better conduit than SVG. Furthermore, technically, the advantage of using RA is the fact that it is not associated with increased rate of sternal wound infection as in LIMA or RIMA use in patients with diabetes.

Evidence for using GEA as a conduit for CABG is promising but lacking. The GEA graft does not have many contraindications to grafting and has a good flow capacity. Additionally, the use of skeletonised grafts has significantly improved previous reported patency rates (66.5% *vs*. 90.2%) at eight years^[^^16^^,^^34^^]^. Nevertheless, a recent meta-analysis did not support this optimistic result and concluded that of all conduits used for CABG, GEA had the highest rates of complete and functional graft occlusion^[^^32^^]^. 

Importantly, however, there is a lack of research validating the patency of these grafts in redo CABG, however the current literature suggests that these grafts maintain similar patency rates as they do in a primary CABG. An example of this includes a large cohort study that showed a significant difference in the 20-year survival and hospital mortality rate in LIMA grafts when compared to SVG in those who had previously undergone CABG (2.2% *vs*. 6.5%, 32% *vs*. 18%, respectively; *P*<0.001)^[^^35^^]^.

**Fig. 2:**
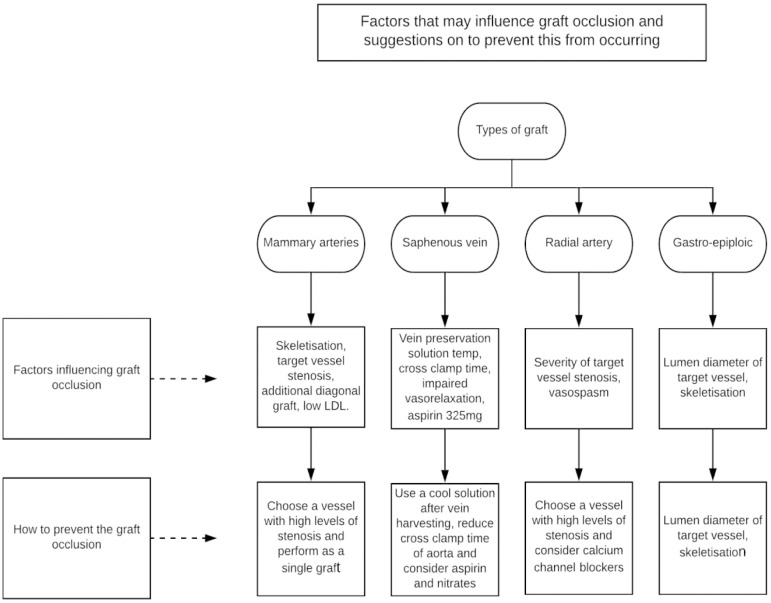
Methods of minimizing graft occlusion. LDL=low-density lipoprotein

Apart from the choice of graft itself, certain factors can predispose grafts to occlusion. Interestingly, a number of papers found that an increased level of cholesterol was not an independent predictor of occlusion, while diabetes was^[^^36^^,^^37^^]^. More specific factors that influence graft patency rate after CABG are summarized in Figure 2^[^^36^^,^^38^^,^^39^^]^.

There is no high-level evidence for many of these factors, but location of graft placement and target vessel stenosis are perhaps the best studied factors shown to influence arterial and now venous graft patency rates after CABG^[^^40^^]^. This is demonstrated by increased patency and survival rates of patients whose vessels had smaller diameters, higher levels of atheroma, and longer length of stenosis prior to grafting^[^^40^^,^^41^^]^. This should therefore be considered prior to grafting and the evidence suggests that grafting too early will lead to poorer patient outcomes. More recently, the importance of graft skeletisation, particularly of GEA, has been demonstrated. A comparison of a previous meta-analysis and a recent study has demonstrated that this skeletisation may affect long-term patency rates by up to 30%^[^^7^^,^^32^^]^. Further predictors of vessel occlusion after CABG require further research and the effect of altering these factors also requires more research^[^^36^^,^^38^^,^^39^^]^. This paper provides possible prevention interventions that should be studied further.

The factors mentioned in Figure 2 are presumed to affect redo CABG similarly, however studies have not exclusively investigated these factors for redo CABG. Nevertheless, one study did find that creatinine and peak creatinine kinase-myocardial band were independent predictors of graft occlusion after a redo CABG or PCI^[^^42^^]^. These factors suggest that acute kidney injury is an important factor to regulate in order to increase the patency of grafts in redo vascularisation procedures.

## REDO CABG, INDICATIONS, AND PITFALLS

The most common indicator for a redo CABG is vein-graft failure from occlusion of a SVG^[^^14^^]^. Patients who qualify for a redo CABG should have at least one graftable coronary artery with an ischaemic territory. If the patient is symptomatic, this is a strong indication for redo CABG. Patients that are being considered for redo CABG should have one or more coronary arteries suitable for grafting and supplying a viable myocardium^[^^5^^]^. Based on the European Guidelines for revascularisation, redo CABG should also be considered for patients with “several diseased grafts, reduced left ventricular function, several chronic total occlusions (CTO), or absence of a patent internal thoracic artery”^[^^8^^]^.

The limited indications for redo CABG incorporate its well documented increased surgical complexity and operative mortality. Interestingly, some current literature suggests that the long-term outcomes for patients undergoing a redo CABG are very similar to those for a patient that has just had the first time operation^[^^43^^]^. These outcomes (in-hospital morbidities and mortality and long-term survival) were based on propensity matching comparing redo-CABG (n = 126) and first-time CABG groups (n = 232), and there were no significant differences in reoperation for bleeding, prolonged ventilation, postoperative stroke, or need for dialysis^[^^43^^]^. The overall finding was that re-sternotomy does not affect long-term survival in the CABG population. Therefore, in those patients that do survive after a redo CABG, there is some evidence to suggest they have relatively good long-term outcomes.

Nevertheless, there are numerous operative components that create difficulties in a redo CABG. The re-sternotomy is technically more challenging as the pericardium has been previously opened and structures adherent to the sternum may be vulnerable, there are fewer conduits in a second revascularisation, and patients are often older and have more comorbidities^[^^9^^]^. Moreover, finding and controlling the patient’s internal mammary artery (IMA) grafts can be difficult and if the IMA graft is injured, the consequences are serious. Based on these complications, the indications for a redo CABG are stricter and are based on a more careful consideration of the overall risks and benefit. In certain situations, an alternative repeat revascularisation treatment may be preferable^[^^5^^]^. This largely depends on the individuals and what has caused their need for a second revascularisation, *e.g*., whether it is the native artery or the graft itself that has become occluded.

Despite these complications, the operative mortality for redo CABG decreased from 6.0% in 2000 to 4.6% in 2009^[^^4^^]^. This study does, however, acknowledge that the incidence of postoperative complications such as renal failure and prolonged ventilation are higher in redo CABG patients in comparison to primary CABG^[^^4^^]^. In contrast to the previous studies that investigated long-term patient outcomes, this study specially refers to the short-term postoperative outcomes. At the present time, surgical experience has diminished in importance as a risk factor in redo CABG and the major contributor to a patient’s outcome are the characteristics and comorbidities associated with the patient^[^^5^^]^.

## PCI IN POST-CABG PATIENTS

For a particular cohort of patients, PCI can be an exceedingly useful treatment and may be the only revascularisation option. The use of PCI was explored in a recent study that investigated its use in five patients with a previous CABG due to left main coronary artery disease with partial or total occlusion of one graft^[^^44^^]^. These patients had one ischaemic territory and the option of either CABG or PCI. Surgery was ruled out due to the high surgical risk, therefore all patients underwent PCI. In a five-year follow-up period, there was one death from a non-cardiovascular related cause and the other four patients remained asymptomatic. This concluded that PCI post CABG can be a useful, safe, and successful treatment and can improve patient outcomes in selected cases and in certain small populations.

A second, similar study assessed the incidence, predictors, and outcomes of early PCI post CABG. They found that 4.4% of 554,987 patients developed postoperative in-hospital coronary ischemia as a complication of CABG^[^^45^^]^. For these patients with early symptoms after CABG, in-hospital PCI was a possible alternative treatment option. The outcomes of patients who required in-hospital PCI post CABG were compared with patients who did not. The primary outcome measure in this study was in-hospital mortality, and in the total cohort of 554,987 patients, 14,323 had undergone early PCI. Mortality was significantly higher in these patients compared to those that did not undergo PCI post CABG (5.1% *vs*. 2.7%, *P*<0.001). 71.4% of these early PCIs were performed 24 hours post CABG.

Therefore, although PCI may be effective in treating graft occlusion after CABG, its use early on after CABG should be limited as it has the propensity to increase patient mortality. With exception to these two studies, there is little evidence concerning to the use of PCI specifically after CABG^[^^44^^,^^45^^]^. Consequently, it is necessary for more research to be carried out before precise conclusions are made about the use of PCI post CABG, including the distinct cohorts that should receive this treatment.

## PCI OR REDO CABG

The literature comparing repeat revascularisation through PCI or redo CABG is consistent in suggesting that both methods of revascularisation improve long-term patient mortality and morbidity when utilized during either early or late graft failure^[^^42^^,^^46^^]^. Nevertheless, there is no consistent statistical difference in patient mortality between these two interventions^[^^42^^,^^47^^,^^48^^]^. Redo CABGs have demonstrated some advantage in long-term symptom relief in comparison to PCI, however the main advantage of receiving a redo CABG is the reduced likelihood of needing further revascularisation intervention at later stage. Studies have demonstrated a 20% reduction in target vessel revascularisation when redo CABG is performed^[^^42^^]^. PCI with drug-eluting stents has significantly improved the need for target vessel revascularisation, however this still “isn’t as good as having a redo CABG”. Despite this “edge” CABG has shown, a redo CABG operation has a two to five times the mortality risk in comparison to the primary CABG and a significantly greater mortality profile than the PCI procedure after a primary CABG^[^^49^^]^. Therefore, this has limited the use of redo CABG to patients with multivessel disease or extensive changes in their coronary vasculature. The risk of these patients occluding in future is high, therefore the higher patency rates and reduced need for revascularisation with redo CABG is more beneficial here. Furthermore, certain anatomical features favour a CABG over PCI. If the IMA-LAD graft is not patent, a redo CABG is more successful due to the high success of arterial grafts for this target vessel^[^^35^^]^. It is therefore imperative that advances to limit the risks of redo CABG are developed and explored to decrease the operative mortality. Recent investigation into off-pump CABG and sternal sparing approaches have shown promise in reducing redo CABG mortality^[^^48^^,^^49^^]^. Initial studies into the off-pump technique in redo CABG have demonstrated no significant difference in operative mortality between on- and off-pump procedures. Nevertheless, the length of hospital stay and mechanical ventilation as well as the number of units of blood needed for transfusion were fewer in the off-pump redo CABG cohorts^[^^49^^,^^50^^]^. More recently, the Japanese Association for Thoracic Surgery published a report illustrating a significant advantage in operative mortality when an off-pump technique was used; although subjects were not matched^[^^51^^]^. Similarly, a left posterolateral thoracotomy for the circumflex artery in patients not suitable for PCI avoids many of the risks associated with a re-sternotomy^[^^52^^]^. The benefits of off-pump surgery and minimally invasive surgery in redo CABG operations create an opportunity to successfully increase the indications for redo CABG, reduce operative mortality, and provide better long-term outcomes of revascularisation in comparison to PCI.

**Fig. 3:**
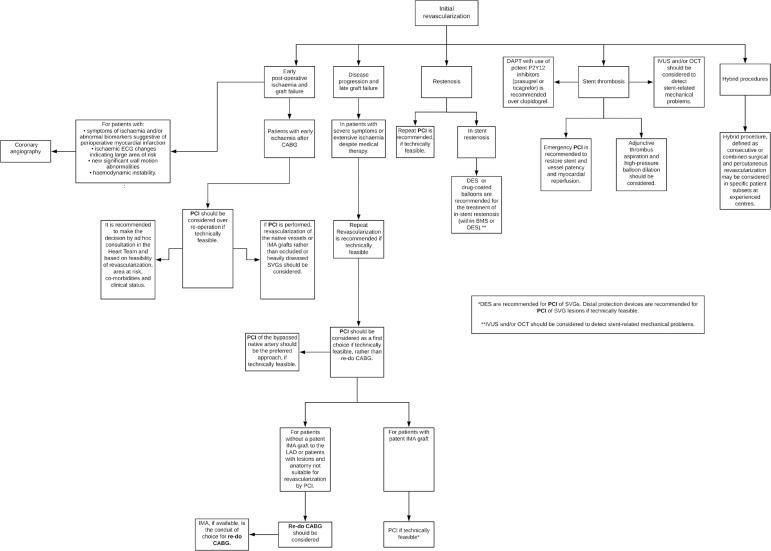
Diagram flow of options for redo revascularization. BMS=bare metal stent; CABG=coronary artery bypass grafting; DAPT=dual antiplatelet therapy; DES=drug eluting stent; ECG=electrocardiography; IMA=internal mammary artery; IVUS=intravascular ultrasound; LAD=left anterior descending artery; OCT=optical coherence tomography; PCI=percutaneous coronary intervention; SVGs=saphenous vein grafts

The decision-making process when selecting PCI or redo CABG as a repeat vascularisation treatment has to be considered carefully, however the guidelines used to make this decision are predominantly based on a patient’s condition and morbidities as opposed to age^[^^2^^]^. Figure 3 is a summary diagram of options for redo revascularization in patients who requires further intervention following initial revascularization. Nevertheless, recent research suggests that the patients’ age may modify the effectiveness of CABG and PCI on preventing further cardiac events; where CABG was favoured at older ages and PCI at younger ages^[^^53^^]^. However, there is a lack of evidence to support any recommendations on which procedure to choose for redo revascularisation depending on age, as such, this has not yet been addressed in coronary revascularization guidelines^[^^2^^,^^53^^]^.

Finally, conservative management may have a role in early and perioperative graft failure post CABG as opposed to acute revascularisation, however, it is generally accepted that these patients will need procedural intervention, and further clinical studies are required to clarify and optimise the use of conservative treatment in the primary graft failure cohort^[^^54^^]^.

## CONCLUSION

Patients that undergo redo CABG are less likely to develop comorbidities associated with revascularisation, but the operative mortality is higher and long term-survival rates are similar in comparison to PCI. Therefore, there is a need for further research into the role of redo CABG in the current advanced practice of PCI.

**Table:** 

Authors' roles & responsibilities	
TEKO	Substantial contributions to the conception or design of the work; or the acquisition, analysis, or interpretation of data for the work; drafting the work or revising it critically for important intellectual content; final approval of the version to be published
KM	Substantial contributions to the conception or design of the work; or the acquisition, analysis, or interpretation of data for the work; drafting the work or revising it critically for important intellectual content; final approval of the version to be published
AH	Substantial contributions to the conception or design of the work; or the acquisition, analysis, or interpretation of data for the work; drafting the work or revising it critically for important intellectual content; final approval of the version to be published
ADM	Substantial contributions to the conception or design of the work; or the acquisition, analysis, or interpretation of data for the work; drafting the work or revising it critically for important intellectual content; final approval of the version to be published
