# ERRATUM

**DOI:** 10.21470/1678-9741-2019-0206e

**Published:** 2025-04-17

In the article “Redo Coronary Artery Bypass Grafting in the era of Advanced PCI”, DOI https://doi.org/10.21470/1678-9741-2019-0206, published in the Brazilian Journal of Cardiovascular Surgery, 37.4, the author’s degree for Kellan Masharani was incorrectly listed as MD.

In the published article, the information was: Ter-Er Kusu-Orkar^1^, MD; Kellan Masharani^1^, MD; Amer Harky^2^, MRCS, MSc; Andrew D Muir^2^, MD

The correct information is: Ter-Er Kusu-Orkar^1^, MD; Kellan Masharani^1^; Amer Harky^2^, MRCS, MSc; Andrew D Muir^2^, MD

BJCVS apologizes for this error that has now been corrected.

